# Aptamer and Electrochemical Aptasensor towards Selenate Ions (SeO_4_^2−^)

**DOI:** 10.3390/ijms25126660

**Published:** 2024-06-17

**Authors:** Anna Szymczyk, Martyna Popiołek, Dominika Baran, Marcin Olszewski, Robert Ziółkowski, Elżbieta Malinowska

**Affiliations:** 1 Chair of Medical Biotechnology, Faculty of Chemistry, Warsaw University of Technology, Stanisława Noakowskiego 3, 00-664 Warsaw, Poland; anna.szymczyk.dokt@pw.edu.pl (A.S.); martyna.popiolek.stud@pw.edu.pl (M.P.); dominika.baran2.stud@pw.edu.pl (D.B.); elzbieta.malinowska@pw.edu.pl (E.M.); 2 Doctoral School, Warsaw University of Technology, Plac Politechniki 1, 00-661 Warsaw, Poland; 3 Chair of Drug and Cosmetics Biotechnology, Faculty of Chemistry, Warsaw University of Technology, Koszykowa 75, 00-664 Warsaw, Poland; marcin.olszewski@pw.edu.pl; 4 Centre for Advanced Materials and Technologies CEZAMAT, Warsaw University of Technology, Poleczki 19, 02-822 Warsaw, Poland

**Keywords:** aptamer, selenate ion, electrochemistry, aptasensor, methylene blue

## Abstract

Selenium is an essential inorganic compound in human and animal nutrition, involved in the proper functioning of the body. As a micronutrient, it actively contributes to the regulation of various metabolic activities, i.e., thyroid hormone, and protection against oxidative stress. However, Se exhibits a narrow concentration window between having a positive effect and exerting a toxic effect. In higher doses, it negatively affects living organisms and causes DNA damage through the formation of free radicals. Increased reactivity of Se anions can also disrupt the integrity and function of DNA-repairing proteins. As the permissible concentration of Se in drinking water is 10 µg/L, it is vital to develop sensitive and robust methods of Se detection in aqueous samples. In this study, for the first time, we proposed a selective aptamer for selenate ion detection, chosen following the SELEX process, and its application in the construction of an electrochemical aptasensor towards SeO_4_^2−^ ions. Measurement conditions such as the used redox marker and pH value of the measurement solution were chosen. The proposed aptasensor is characterized by good selectivity and an LOD of 1 nM. Conditions for biosensor regeneration and storage were also investigated in this research.

## 1. Introduction

Selenium exists in all the components of the environment, including rocks, soil, plants, and water. Wastewater discharged from mining, petrochemical agricultural, or metallurgical activities contributes to elevated Se levels in groundwater, which can quickly reach levels toxic to fish and wildlife [1]. Selenium can be found in four different oxidation states as elemental selenium Se(0), as selenide Se(-II) in organic form and in inorganic forms as oxyanions, selenite (SeO_3_^2−^) (-IV) and selenate (SeO_4_^2−^) (-VI). Inorganic forms of selenium species are considered more toxic than their organic forms due to substantially better water solubility and environmental mobility [2]. Selenite Se(IV) is known as the most toxic and selenate Se(VI) as the most bioavailable and soluble compound in the oxidizing environment [2,3]. However, because of its high capacity for bioaccumulation, the substantial harmfulness of selenate anions is also described [4,5].

For the speciation analysis and determination of anionic selenium forms in water, many spectroscopic techniques have been developed, including inductively coupled plasma mass spectrometry (ICP-MS) [6,7,8], inductively coupled plasma optical emission spectroscopy (ICP-OES) [9], hydride generation atomic absorption spectrometry (HGAAS) [10,11], nuclear magnetic resonance imaging (NMRI) [12], neutron activation analysis [13] and fluorometry [14]. Separation techniques such as high-performance liquid chromatography (HPLC) [15], solid-phase extraction (SPE) [16], ion-exchange chromatography (IEC) [17] and capillary electrophoresis [18] have also been employed to improve the performance of the existing selenium detection methods. Unfortunately, a laboratory equipped with expensive analytical devices is indispensable for carrying out such analyses and labor-intensive sample preparation. These methods are also time-consuming and require highly trained personnel.

An alternative to the approaches mentioned above is the employment of electrochemical techniques, which are characterized by simplicity, high sensitivity, and the capacity of miniaturization for on-site applications. To date, stripping voltammetry with the use of modified solid electrodes has been the most widely used approach. Various electrode modifiers, including mercury films [19], boron-doped gold modification of diamond electrodes [20] as well as direct detection with platinum and gold electrodes, microband electrode array [21] and a rotating gold electrode [22], have typically been employed for Se determination. Also, the use of nanomaterials opens up new possibilities in the detection of selenium anions. For this purpose, nitrogen-doped graphene [23], reduced graphene oxide [24], Au/ZnO-nanocomposite-decorated ITO electrodes [25], poly(1-aminoanthraquinone)/multiwall carbon nanotubes [26] and Mn_3_O_4_^−^ chitosan nanocomposite [27] have been used. The methods mentioned above offered the possibility of determination at satisfactorily low concentration levels, i.e., below 10 µg/L (WHO limit [28]). However, the selectivity of voltametric methods can often be unsatisfactory. Therefore, it is desirable to develop electrochemical sensors with enhanced selectivity, e.g., by designing and introducing selective bioreceptors with an affinity for selenium species. Only a few such attempts have been described in the literature [29,30]. One of them is the approach proposed by Motlagh et al., who developed an enzymatic gold nanodendrite biosensor. Selenate reductase immobilized on the electrode surface reduces selenate to selenite ion, which, as an electroactive compound [31], is then detectable by CV and DPV voltammetry [29]. A similar approach is described by the same author in [30], where, instead of a pure enzyme, the bacterial strains capable of selenate reduction were used. A different approach in biosensor development, instead of reaction catalyzed by specific enzymes, is the use of receptors that express high affinity toward a given analyte, selenate ion (SeO_4_^2−^), and its subsequent binding in the receptor layer. One example of such receptors is nucleic acid strands or, more specifically, selected sequences of nucleic acids, called aptamers. Thanks to the abundance of DNA nucleotide functional groups as aptamer building blocks, aptamers can interact according to various mechanisms with a wide range of targets, including small molecules such as inorganic anions. Another advantage of oligonucleotide receptors is the possibility of their design and synthesis in vitro, opposite to protein receptors, and their activity insensitivity even toward toxic compounds. Moreover, analyte-triggered conformational changes can be a source of analytical signal, which opens vast possibilities for the employment of aptamers in various detection strategies, including electrochemical detection. Despite the apparent advantages of aptamers as molecular receptors, the design of aptasensors for selenate (SeO_4_^2−^) anions has not yet been described. This may be explained by the fact that the selection of aptamers for ions and small molecules is challenging. The small molecular weight of such targets and a huge difference in size in comparison to the oligonucleotides significantly hinder the SELEX (Systematic Evolution of Ligands by EXponential enrichment) process. Particular difficulties arise at the step of the separation of unbound DNA sequences from target–aptamer complexes that differ only slightly in mass and general properties [32]. Another challenge in the selection of aptamers for small molecules is the lack of epitopes or functional groups available for strong aptamer binding and the same lower affinity of the aptamers to small target molecules in comparison to larger analytes [33]. Nevertheless, after the identification of the above hindrances, there are also several approaches to solving such problems (e.g., reversible aptamer candidate immobilization on magnetic nanoparticles, which increases the efficiency of the separation of unbound nucleic acid strands) [32] and allowing for the selection of new aptamers offering high affinity toward ions.

In this study, for the first time, we report the sequence of DNA aptamer as novel receptors towards SeO_4_^2−^ ion, chosen following the SELEX process, and we describe their introduction for the construction of an electrochemical aptasensor towards selenate ion. The developed biosensor allows for the direct detection of selenate ions in aqueous samples. We believe that the high selectivity and sensitivity of the developed aptasensor are determined by the high-affinity aptamer to the given selenium ion, as well as the proposed detection strategy based on the use of methylene blue (MB) as an electrochemical redox marker. The performed studies also cover the optimization of the sensing conditions, with a focus on the selection of measurement medium composition suitable for the detection of the biosensor response. Conditions for biosensor regeneration and storage were also investigated.

## 2. Results and Discussion

**Figure 1:**
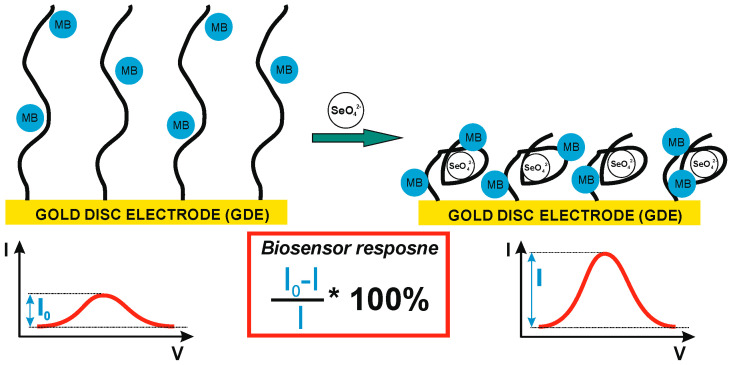
Schematic illustration of the biosensor response mechanism and the equation used for biosensor response calculation.

To date, several aptamers toward inorganic ions and electrochemical aptasensors using such aptamers within receptor layers have been described [34,35,36]. This included mercury (Hg^2+^), lead (Pb^2+^), potassium (K^+^), uranyl (UO_2_^2+^), silver (Ag^+^) or cadmium (Cd^2+^). In all cases, the interactions between the given ion and ssDNA aptamer(s) resulted in its binding and retention in the biosensing layer. Several proven mechanisms are responsible for this, including G-quadruplex stabilization by Pb^2+^ and K^+^ [37], cytosine–cytosine mismatch stabilization by Ag^+^ [38], covalent bond formation between thymine- and guanine-rich probes and Cd^2+^ [39], coordinate bond formation between the phosphate backbone and UO_2_^2+^ [40], and thymine–thymine mismatch stabilization by Hg^2+^ [41]. In spite of the above, the standard procedure for new aptamer selection involves obtaining a pool of nucleic acid sequences of increasing affinity toward a given analyte in subsequent rounds (between 6 to 12) [42]. The above procedure, however, was developed for proteins, which, compared to inorganic ions, are analytes of rather sizable dimensions, 1–100 nm [43] and 0.027–0.3 nm [44], respectively. To be able to obtain aptamer sequences for such small analytes as inorganic ions, the VENNMultiplex™ mode of SELEX can be used [45]. In the presented studies, the aptamer selection process, consisting of 12 rounds, allowed for defining a sequence of high specificity towards SeO_4_^2−^ ions. It was, however, necessary to further prove that this sequence could be used in the biosensor layer of the electrochemical aptasensor as a receptor towards selenate ions. The biosensor construction was based on a typical self-assembled monolayer setup [46]. In order to be able to immobilize the investigated aptamer on the gold disc electrode surface, it was modified with a thiol group. After its deposition on the gold substrate, the blocking 6-mercapto-1-hexanol was immobilized to cover the electrode area not occupied by ssDNA [46,47]. For electrochemical signal generation, the redox marker was used, freely available in the solution. The mechanism of detection of the developed electrochemical aptasensor, confirmed during the presented studies, is shown in Figure 1, together with the equation for biosensor response calculation.

Briefly, as the redox indicator (the only electroactive compound in the used potential range, approximately from −0.2 to 0.8 V) is freely available in the sample, the electrochemical biosensor response will depend on the change in the efficiency of its oxidation or reduction. This efficiency, in turn, depends on the possibility and easiness of reaching the electrode surface. As it changes together with binding the analyte by the receptor layer, the observed changes in redox current are translated into the biosensor response (Figure 1). In the presented studies, ultimately, the cationic marker, methylene blue, was chosen, and the observed tendency in redox current changes is shown in Figure 1. However, as several such indicators are available, which differ in structure and charge and share a mechanism of interaction with DNA strands, this ultimately translates into smaller or larger changes in redox reaction efficiency triggered by binding of the analyte (e.g., selenate ion) by the receptor layer; initially two such indicators were investigated as the source of the signal for the biosensor response [46]. These were (i) methylene blue, an aromatic compound exhibiting a positive charge, and (ii) an equimolar mix of ferro-/ferricyanide with a negative charge. Moreover, the evaluation of the biosensor response registered for such different redox markers may point to its source.

**Figure 2:**
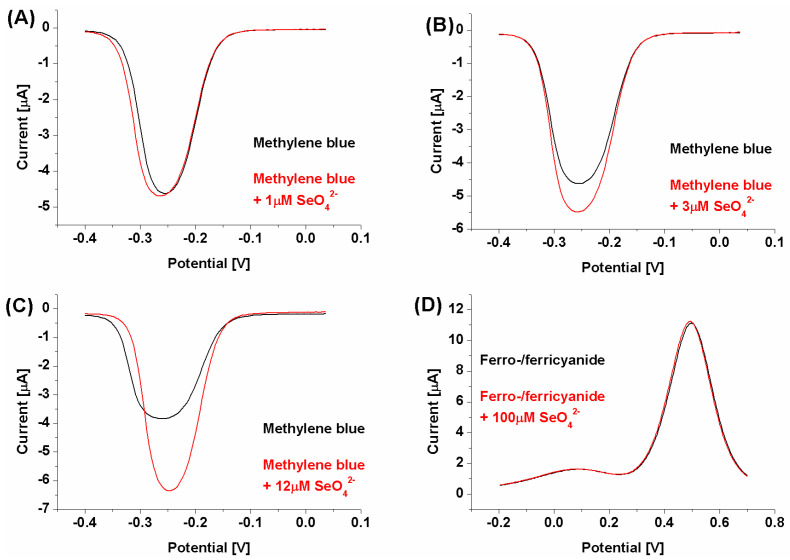
SWV voltammograms. (**A**–**C**): Biosensor response registered with methylene blue as a redox marker with an increasing selenate ion concentration (from 1 to 12 μM); (**D**): biosensor response registered with ferro-/ferricyanide as a redox marker with a selenate ion concentration of 100 μM.

The initial experiments were conducted at pH 7.0 (Figure 2), which is optimal for DNA strands [48,49] and has a negligible influence on the selenate ion [50]. It should be remembered that depending on the medium composition and properties (e.g., pH), the analyzed ion could be present in a different form than initially assumed. One of the best examples of the measurement environment’s influence on the form of analyzed anion, and the same on the obtained results, could be mercury ion (Hg^2+^) [51,52,53], where, depending on medium pH and its composition, mercury could be present as HgCl_2_, Hg(OH)_2_, HgClOH, HgOHCO_3_^−^, or complexed by the components of the measurement solution [52]. Luckily, selenate anions (SeO_4_^2−^) are quite stable across a broad pH range (pH between 3.5 and 14.0). Only below pH 3.0 is it protonated and changes into HSeO_4_^−^ [50]. Similar changes could also be observed for the receptor layer. DNA molecules are stable in the pH range from 4.0 to 9.0 [48,49], but in more extreme values, they are susceptible to pH-dependent destabilization. Below pH 3.5, DNA loses purine bases (adenine and guanine) in the so-called depurination process, and below pH 2.0, it also loses its polyanionic character due to the protonation of the phosphate backbone [48,49,54]. In turn, for pH higher than 9.0, dsDNA is prone to alkaline denaturation due to the abundance of hydroxide ions, which break the hydrogen bonds between DNA strands (remove hydrogen ions from the base pairs of DNA). Nonetheless, as can be seen in Figure 2, the biosensor response at pH 7.0 toward selenate ion (calculated according to the equation presented in Figure 1) was obtained only when methylene blue was used as the redox marker (square wave voltammetry anodic or cathodic scans were chosen based on higher current changes).

**Figure 3:**
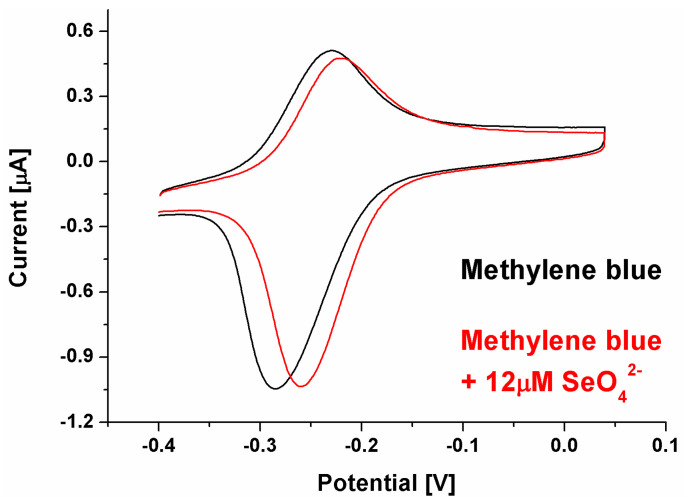
CV voltammogram for biosensor response registered with methylene blue as a redox marker with a selenate ion concentration of 12 μM.

This might result from the electrostatic interactions present in the receptor layer. In the case of the cationic redox marker (methylene blue), its attraction by a negatively charged phosphate DNA backbone moves the net receptor layer charge toward being more positive, which may make the selenate anions approach the electrode surface. However, binding SeO_4_^2−^ by aptamer strands and its retention in the biosensing layer should diminish the current registered for the aptasensor after the recognition process due to the formation of possible steric hindrances for redox markers approaching the electrode surface [55], which were not observed to increase, as shown in Figure 2. Nonetheless, in the literature, there are also several examples of electrochemical signal increase after analyte binding by the receptor layer [56,57]. Similarly to these reports, we conclude (which is also pictured in Figure 1) that the change in spatial aptamer shape after selenate ion binding leads to more space in the receptor layer for the redox marker to approach the electrode surface. We also conclude that because of exactly the same electrostatic hindrances, no aptasensor response was registered when the anionic redox marker, equimolar mix of ferro-/ferricyanide, was used (even for substantially higher selenate ion concentration, 100 μM, compared to assays with methylene blue, 1–12 μM). As described above, the electrostatic mechanism of attraction or repulsion of the investigated redox marker could also be confirmed by increased reversibility of registered redox reaction after SeO_4_^2−^ ion binding by the receptor layer (ΔE changes from 54 to 38 mV); see Figure 3.

**Figure 4:**
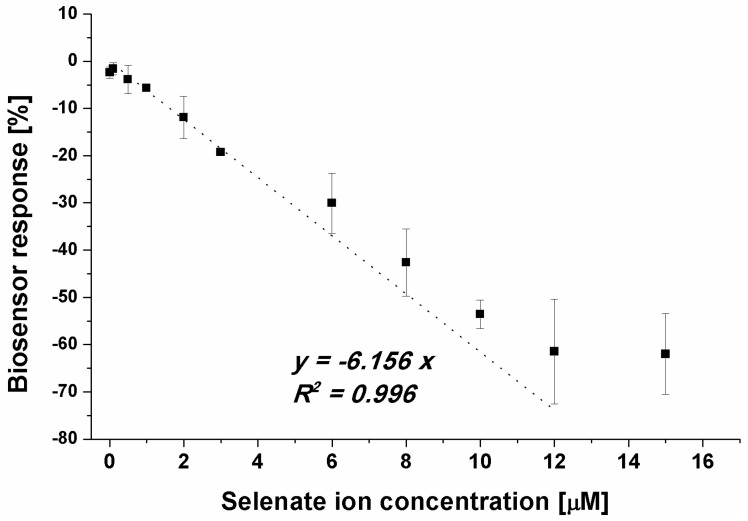
Dependency between selenate ion concentration and calculated biosensor response (result obtained for SWV experiments, *n* = 4).

Because of the registered aptasensor response for selenate ion in an assay with methylene blue as a redox marker, we evaluated the biosensor response for a given concentration range of selenate ion (Figure 4).

**Figure 5:**
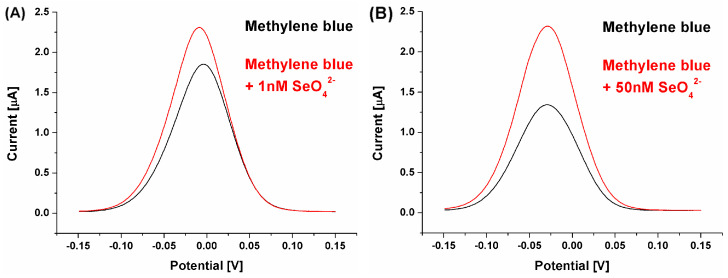
SWV voltammograms for biosensor response registered with methylene blue (pH 4.0) as a redox marker and for selenate ion concentration at the level of (**A**) 1 nM and (**B**) 50 nM.

Although it was possible to observe the dependency between the biosensor response and SeO_4_^2−^ concentration in the sample (Figure 4), the obtained LOD at the level of 1 μM (linear range from 1 to 12 μM) was not satisfactory as the limit for selenium presence in water samples specified by the WHO is at the level of 10 µg/L [28], which gives approximately 0.127 μM. As the specified WHO limit and the obtained LOD differ by 10-fold, an attempt was made to lower the detection limit. Based on the above results, we attempted to move the overall receptor layer charge toward more positive, which should increase the efficiency in SeO_4_^2−^ anion approximation to the electrode surface. In the presented studies, it was realized by lowering the pH of the measurement medium to pH 4.0. Such a change results in the protonation of the last element of the receptor layer, 6-mercapto-1-hexanol, which, at higher pH values, exhibits a partially negative charge (originating from –OH moieties) and is protonated near pH 5.0 [58]. As was already stated, the selenate ion is stable in the broad pH range [50] and retains its form, SeO_4_^2−^. For single-stranded DNA, with our aptamer used as a receptor, depurination takes place below pH 3.5, which allowed us to conduct experiments at pH 4.0 (Figure 5).

**Figure 6:**
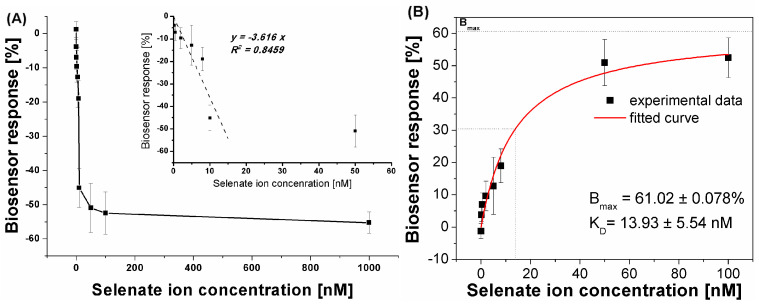
(**A**) Dependency between selenate ion concentration and calculated biosensor response (result obtained for SWV experiments, *n* = 4); (**B**) plot of the absolute aptasensor electrochemical responses (transformed to positive values versus calculated with the equation presented in Figure 1) in the function of analyte concentration for the determination of equilibrium dissociation constant (K_D_). Black points represent experimental points; red curve represents fitting through non-linear regression according to the “one-to-one” kinetic model.

The change in the overall receptor layer charge (toward more positive values) could be observed already for the redox reaction of cationic methylene blue. As its concentration was not changed (50 μM) between assays conducted at pH 4.0 and 7.0, and the registered currents were two-times smaller than for pH 7.0, it can be concluded that its electrostatic attraction by the receptor layer was of significantly lower efficiency. Also, the methylene blue redox potential was moved toward more positive values, from approximately −0.25 V for pH 7.0 to approximately −0.025 V for pH 4.0, which additionally points to changes in its interactions with the receptor layer. However, what is significantly more important is the fact that the calculated biosensor response was high even for a significantly lower selenate ion concentration than during measurements at pH 7.0. This, in turn, could originate from the fact that, in these conditions, the receptor layer negative charge originates only from the immobilized ssDNA aptamer strands, which approximates, at least theoretically, conditions where the aptamer sequences were selected in the depth of solution during the SELEX process. Nonetheless, the change in the pH value of the measurement environment resulted not only in a change in registered voltammograms (methylene blue redox reaction) but also in the obtained analytical parameters of the aptasensor toward the analyte (SeO_4_^2−^). Although we expected an increased aptasensor response toward the analyzed ion, the obtained LOD (approximately 1 nM) was far below the above-mentioned detection limits specified by the WHO [28] (Figure 6A).

This made us evaluate the strength of the binding affinity between the obtained receptor layer with the developed aptamer and the selenate ion by calculation of the dissociation constant (K_D_). Assuming a simple aptamer–analyte interaction at equilibrium according to the “one-to-one” kinetic model and good interaction stability, it was possible to numerically determine the equilibrium dissociation constant (K_D_) [59]. This parameter reflects the binding affinity of the target to the aptamer molecule. As can be seen in Figure 6B, the K_D_ value at the level of 13.9 nM indicates the high ability of aptamer binding sites to form complexes with the detected analyte, even at its low concentrations in the sample.

**Figure 7:**
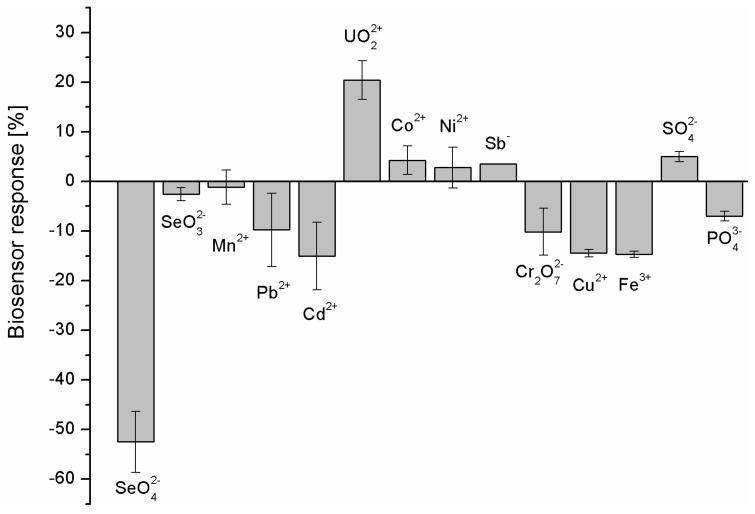
Selectivity studies of as-prepared electrochemical aptasensor. All ions were at the level of 100 nM and the sensor incubation in the sample was conducted for 30 min (*n* = 4).

The calculated dissociation constant proved the high affinity of the as-prepared biosensing layers toward selenate ions in the used measurement conditions. This was further confirmed by the selectivity studies (Figure 7), where all analyzed ions were at the level of 100 nM.

As can be seen in Figure 7, the highest aptasensor response was obtained for selenate ion (−52.48 ± 6.00%) followed by Cd^2+^ (−15.04 ± 6.80%) and Fe^3+^ (−14.69 ± 0.63%). The lowest biosensor responses were registered for Ni^2+^ (2.80 ± 4.14%) and Sb^−^ (3.50 ± 0.00%). The clear decrease in the registered methylene blue reduction current after biosensor incubation in the sample (different biosensor response) was registered for UO_2_^2+^ (20.41 ± 3.91%). These could indicate that strong binding of anions or cations in the receptor layer changes the overall charge of such a layer. In the case of UO_2_^2+^ cation, which forms coordination bonds with phosphates present in the DNA backbone, the charge becomes more positive, which diminishes the electrostatic attraction of the positive redox marker, methylene blue, the same reducing the registered current. As we can see in Figure 7, a different biosensor response is observed for selenate anions. In this case, the current increases, which may suggest the increase in the negative charge deposited at the electrode surface (increased attraction of cationic redox marker, methylene blue) and increased efficiency in its redox reaction. Additionally, importantly, the aptasensor response toward SeO_3_^2−^ was negligible.

**Figure 8:**
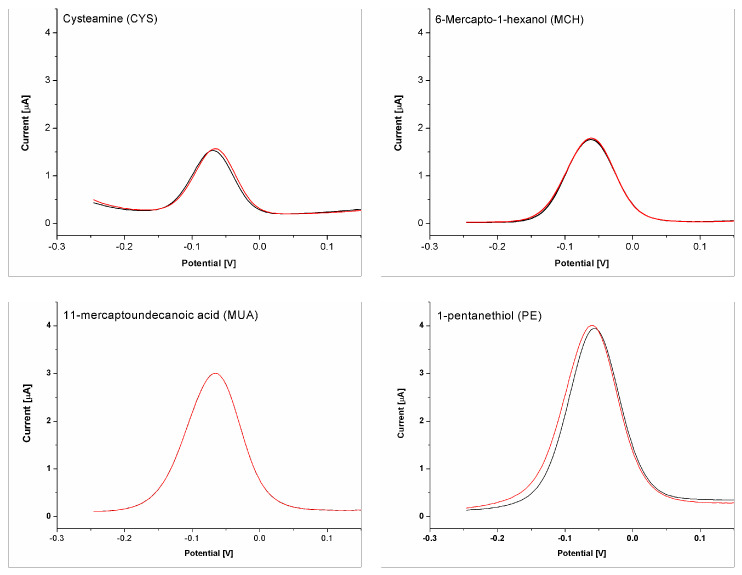
Response of the electrodes modified with appropriate electrode blocking agent (cysteamine, 6-mercapto-1-hexanol, 11-mercaptoundecanoic acid or 1-pentanethiol) for measurement solution containing only methylene blue (black line) and for methylene solution with selenate ion addition (100 nM) (red line).

In order to further confirm that the observed biosensor response results from the selenate ion binding by the aptamer, and not with any other interactions that could take place within the receptor layer, we modified electrodes only with different blocking agents (EBAs). This allowed us to obtain different surface properties: (i) hydrophilic with neutral charge for 6-Mercapto-1-hexanol (MCH); (ii) hydrophilic with neutral-to-negative charge for 11-mercaptoundecanoic acid (MUA; pKa of pH~4); (iii) hydrophilic with positive charge for cysteamine (CYS); (iv) hydrophobic with neutral charge for 1-pentanethiol (PE). Then, such prepared electrodes were used in selenate ion detection as if it were a typical aptasensor. The registered “biosensor” response is presented in Figure 8, together with example voltammograms. As can be seen, the registered differences in the “response” toward selenate ion (a few percent of changes) for every EBA used are within the margin of error and do not indicate the selenate ion detection. The only differences were registered for methylene blue redox current intensity after signal stabilization, where, for a positively charged (CYS) electrode, the lowest signal was obtained, then, together with increasing the negative charge density at the electrode surface, the registered current also increases (MCH and MUA). The highest current was registered for PE, which most likely results from the lipophilic character of the electrode surface, which could more efficiently attract the methylene blue. However, different currents registered for different EBA used could lead to diminishment or increase the electrochemical aptasensor response toward selenate ion, which will be subjected to other studies by us. However, importantly, the above results clearly indicate that the change in current after subjection of the prepared aptasensor to the selenate ions comes almost only from the binding of selenate ions to the aptamer, not from any other interactions that could take place within the receptor layer.

**Figure 9:**
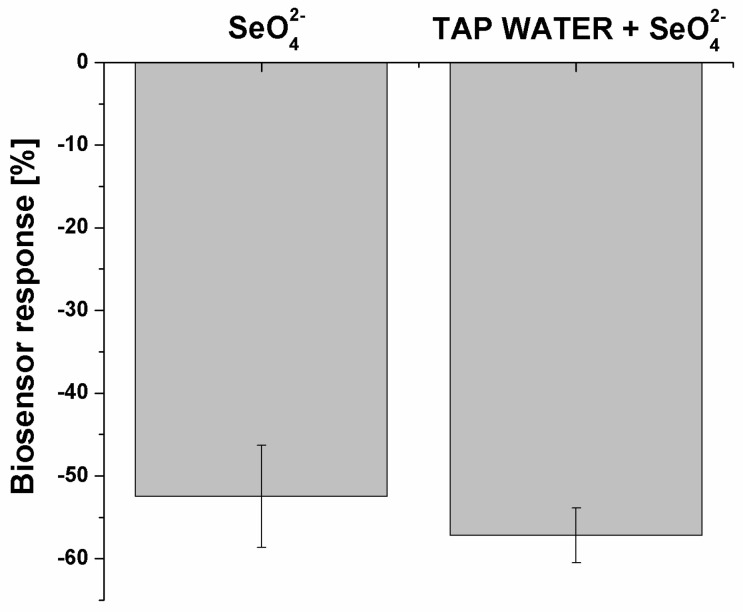
Real sample analysis with the use of the prepared biosensor. The selenate ion concentration was 100 nM (*n* = 4).

Both the presented selectivity and the low dissociation constant motivated us to also evaluate the developed biosensor response for selenate ion in the real sample. This was tap water, as we initially followed WHO regulations related to the selenate limits in drinking water (Figure 9).

The tap water was diluted 1:1 (*v*/*v*) with measurement solution (twice concentrated) and spiked with 100 nM SeO_4_^2−^. As was previously mentioned, the limit specified by the WHO for selenium concentration in drinking water is at the level of 0.01 mg/L (0.127 μM) [28]. As can be seen in Figure 9, the developed aptasensor response was only slightly higher for the spiked real sample (−57.19 ± 0.03%) than for the laboratory sample (−52.48 ± 0.06%).

**Figure 10:**
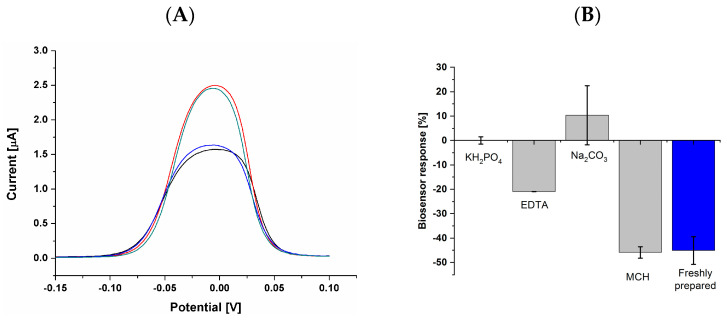
(**A**) Aptasensor regeneration studies. Black line: before selenate ion detection; red line: after 30 min of electrode incubation in selenate ion solution (100 nM); blue line: after 20 min of aptasensor incubation in 0.5 M Na_2_CO_3_ at pH 10.5 (regeneration); green line: after 30 min of electrode incubation in selenate ion solution (after regeneration process); (**B**) aptasensor response (50 nM selenate ion concentration) after one week of storage in the specific conditions (blue bar: response obtained with freshly prepared biosensor).

Further studies were dedicated to (i) the evaluation of the possibility of biosensor regeneration and its subsequent use in selenate ion concentration determination (Figure 10A) and (ii) the evaluation of the biosensor stability during one week of storage in chosen conditions (Figure 10B). The first mentioned experiments involved 20 min of aptasensor incubation in one of the following solutions; 5 mM EDTA, pH 7.5 (as a standard complexing agent that proved its applicability in multiple studies [51]); 1 M KH_2_PO_4_, pH 4.5 (used in the presented studies as solution for DNA immobilization); solution of 10 mM TRIS, 50 mM KCl and 20 mM MgCl_2_, pH 7.0 (used in various studies as a typical DNA/DNA hybridization buffer [47]); and 0.5 M Na_2_CO_3_, pH 10.5 (with a significantly changed pH and presence of CO_3_^2−^ ions, which, according to the literature, can compete with selenate ions during its removal with magnetite from granitic groundwater [60]).

From the analyzed condition, the regeneration process took place only for sodium carbonate, and the appropriate current change after subsequent selenate ion determination was obtained (Figure 10A). The investigations on biosensor storage were conducted in 1 M pf KH_2_PO_4_ at pH 4.5, 5 mM of EDTA at pH 7.5, (used in the presented studies as solution for DNA immobilization), 0.5 M of Na_2_CO_3_ at pH 10.5, and water solution of 2 mM of 6-mercapto-1-hexanol (MCH). From the analyzed conditions, only the biosensor that was kept in 2 mM of MCH was able to obtain similar results for the determination of 50 nM selenate ion at a similar level to the freshly prepared biosensor (Figure 10B).

Nonetheless, taking into account the obtained results, aptasensor response dependency to selenate ion concentration (Figure 6A) and to a real sample (Figure 9), low dissociation constant of as-prepared receptor layer (Figure 6B) (K_D_ at the level of 13.9 nM) and obtained selectivity (Figure 7), we believe that there is the possibility of using the described aptamer sequence in the construction of biosensing layers toward SeO_4_^2−^ anion.

## 3. Materials and Methods

### 3.1. Reagents

2-Amino-2-(hydroxymethyl)-1,3-propanediol—Trizma^®^ base (TRIS), 3-(N-morpholino)propanesulfonic acid (MOPS), sodium dihydrogen phosphate (NaH_2_PO_4_), potassium dihydrogen phosphate (KH_2_PO_4_), potassium chloride (KCl), sodium hydroxide (NaOH), 6-mercapto-1-hexanol (MCH), methylene blue (MB), lead(II) nitrate (Pb(NO_3_)_2_), cadmium chloride (CdCl_2_), copper(II) nitrate (Cu(NO_3_)_2_), iron(III) chloride (FeCl_3_), uranyl acetate (UO_2_(CH_3_COO)_2_), cobalt(II) sulfate hexahydrate (CoSO_4_·6H_2_O), potassium chromate (K_2_CrO_4_), manganese (II) chloride (MnCl_2_), nickel nitrate (Ni(NO_3_)_2_), sodium selenate (Na_2_SeO_4_), and potassium antimony (III) tartrate hydrate (C_8_H_4_K_2_O_12_Sb_2_·H_2_O) were purchased from Merck (Darmstadt, Germany). Sulfuric acid (H_2_SO_4_) and hydrogen peroxide (H_2_O_2_) were purchased from POCh (Gliwice, Poland). Potassium ferricyanide (K_3_[Fe(CN)_6_]) and potassium ferrocyanide (K_4_[Fe(CN)_6_]) were purchased from Fluka (Buchs, Switzerland). All reagents were used without further purification. All solutions were prepared with Milli-Q water.

DNA aptamer specificity towards selenate ions was identified through SELEX process by Basepair Biotechnologies (Pearland, TX, USA) with the use of the VENNMultiplex™ technique dedicated to low molecular targets. In this SELEX process type, aptamer candidates were hybridized to the complementary sequences immobilized on the surfaces of magnetic nanoparticles. After each round, aptamer candidates bound with the selenate ion were removed from the nanoparticles and stayed in the supernatant after the magnetic sample separation. Application of the SELEX procedure included the use of VENNMultiplex™ for at least 12 rounds, which was followed by next-generation sequencing and bioinformatics studies. Based on results, the 32-nucleotide single-stranded DNA aptamer probe E1 (desalted, HPLC purified) specific towards selenate ions and additionally containing a disulfide group was purchased from Metabion, Planegg, Germany. The sequence was as follows: 5′–OH–C_6_–S–S–C_6_- TAT GAC ATT GTG ACG AAC TCC TCA CTA GAC CG. Aptamer stock solutions (100 μM) were prepared with a water-molecular biology reagent (Merck, Germany) and stored in a −20 °C freezer before use.

### 3.2. Solutions

The solutions used in the presented experiments were as follows: 50 mM MOPS pH 7.0; 5 mM ferri/ferrocyanide in 50 mM MOPS, pH 7.0; 50 μM MB in 50 mM MOPS pH 7.0; 50 μM MB in 50 mM TRIS pH 4.0; twice-concentrated measurement solution in studies conducted for real sample analysis (100 μM MB in 100 mM TRIS pH 4.0); 5 mM ferri/ferrocyanide in 0.1 M KCl; 1 M H_2_SO_4_, 0.1 M H_2_SO_4_, 1 M NaOH, piranha solution (H_2_SO_4_:H_2_O_2_ (3:1) (*v*/*v*)), 1 M KH_2_PO_4_, pH 4.5; water solution of 2 mM 6-mercapto-1-hexanol. If necessary, redox indicator solutions were spiked with appropriate salts.

### 3.3. Gold Electrodes Cleaning and Modification

The electrochemical measurements were carried out with a conventional three-electrode system consisting of a gold disk electrode (CH Instruments, Bee Cave, TX, USA), a gold wire as an auxiliary electrode (Sigma Aldrich, Darmstadt, Germany), and Ag/AgCl/1 M KCl reference electrode (Mineral, Warsaw, Poland). The gold disc electrodes were prepared according to [47]. After electrode cleaning, the given concentration (usually 4 μM) of thiolated DNA probe in immobilized buffer solution (1 M KH_2_PO_4_ (pH 4.5)) was deposited at the surface of previously cleaned electrodes and immobilized for a given period of time (e.g., 30 min). Then, the electrodes were washed in DI water and incubated for 2 h in 2 mM MCH. Next, electrodes were rinsed with DI water and kept for 2 min in 100 mM NaOH with 1 M NaCl to remove weakly bound DNA/MCH moieties before the electrochemical measurements.

### 3.4. Electrochemical Measurements

Voltametric measurements were performed with a CHI660A electrochemical workstation (CH Instruments, USA). Cyclic voltammetry (CV) was conducted at a sweep rate of 100 mV·s^−1^, while square wave voltammetry (SWV) was recorded at a pulse amplitude of 25 mV, an increment of 4 mV, and a frequency of 15 Hz. Depending on the pH of the measurement solution, cathodic or anodic scans were taken into account during qualitative and quantitative analyses. This was based on calculations made after each set of experiments. Before measurements, electrodes were incubated in an analyzed measurement buffer for 10 min. Then, a series of SWV measurements were conducted to stabilize the initial receptor layer signal (I_0_).

## 4. Conclusions

Due to human activity, particularly related to natural environment degradation and changes in the global temperature values, the distribution of various pollutants or toxins in the environment changes. This may result in groundwater contamination and consequent bioaccumulation of compounds hazardous to human health in agricultural crops, animals consumed, and in the air we breathe. This, in turn, entails the need for the elaboration of mobile and easy-to-use analytical devices, which could be operated by non-professionals directly in the place of need. One such approach is to use electrochemical sensors and biosensors, which, because of their dimensions, low cost, and ease of integration in more advanced microfluidic devices, make such solutions possible. However, one of the most important parts of such biosensors is the receptor layer and, more precisely, the receptor, which could selectively bind the analyte of choice. The progress in new receptor development is directly connected to the progress in the development of the abovementioned sensors and biosensors and, ultimately, also to the mobile devices capable of precise analysis of the sample in the place of sampling. One example of such receptors is aptamers, single-stranded DNA or RNA oligonucleotides selected during the SELEX process. Herein, we present the 32-nucleotide aptamer sequences, identified during the VENNMultiplex™ mode of systematic evolution of ligands by exponential enrichment. It was further modified with the thiol group at its 5′ end and applied for the first time in the receptor layer of electrochemical aptasensor toward selenate ion. During the presented studies, the measurement conditions were chosen, specifically the redox marker used and pH value of the measurement solution. This, however, makes it possible to elucidate, at least at the very first step, the mechanism of SeO_4_^2−^ ion interaction with the receptor layer and to obtain interesting analytical parameters of the developed electrochemical aptasensor. Especially from the point of view of the level of detection, which was far below the limits described by the WHO (10 μg/L) and selectivity. For elaborated receptor layer and measurement conditions, the dissociation constant was calculated (13.93 ± 5.54 nM), which further indicates the high ability of aptamer binding sites to form complexes with the detected analyte even at its low concentrations in the sample. It should, however, be emphasized that this is by far the first attempt to utilize aptamer-based layers for electrochemical detection of selenate ions, and this paper does not provide an exhaustive list of aptasensor construction examples. We believe that further studies will allow for increased repeatability of the conducted experiments, shorten the analysis, or make it more user friendly. These can be achieved, e.g., by the application of other electrode blocking agents [47,61], optimization of the receptor density within the biosensing layer and measurement solution composition, or application of transducers manufactured in printed electronic technology [51].

## 5. Patents

Authors have patent “DNA aptamer sequence, its use for selective detection of selenium ions, a method of manufacturing an electrochemical aptamer sensor and a method of measurement using it, and an electrochemical aptamer sensor containing an oligonucleotide DNA aptamer sequence”, P.448520 pending to Assignee.

## Data Availability

Data are available on request.
